# Special Issue “Advances in Diagnostics: Applications of Nucleic Acids and Their Analogs”

**DOI:** 10.3390/ijms27020896

**Published:** 2026-01-16

**Authors:** Annalisa Masi, Barbara Pascucci, Maria Moccia

**Affiliations:** Institute of Crystallography, National Research Council, Strada Provinciale 35d, 9, Montelibretti, 00010 Rome, Italy

Nucleic acids and their synthetic analogs have driven modern diagnostics of versatile platforms capable of detecting disease signatures with molecular precision [1]. Classical hybridization assays based on sequence complementarity have been strengthened by peptide nucleic acids (PNAs) and locked nucleic acids (LNAs)**,** which provide higher affinity, improved mismatch discrimination, and resistance to nuclease degradation, enabling sensitive detection in complex matrices [2,3]. Conformation-responsive elements such as aptamers further expand capabilities: they offer antibody-like affinity with superior thermal/chemical stability and batch consistency, making them suitable for portable and point-of-care devices [4,5,6]. In parallel, CRISPR-based detectors exploit programmable recognition and collateral nuclease activity (e.g., Cas13, Cas12) to achieve isothermal signal amplification and very low limits of detection in platforms such as SHERLOCK and DETECTR [7,8]. Finally, diagnostic readouts now extend beyond sequence to epitranscriptomic and epigenetic marks: RNA modifications (e.g., m^6^A, m^5^C) and DNA hydroxymethylation provide insight into cell state and disease. These marks are becoming increasingly measurable owing to advances in selective chemistry, enzymatic labelling, and sequencing/nanopore-based detection methods [9,10,11].

With this perspective, we present the Special Issue “Advances in Diagnostics: Applications of Nucleic Acids and Their Analogs”, a collection that spans experimental molecular methodologies, computational approaches, and pathways toward clinical translation.

Within this framework, the collected articles highlight the advances of DNA diagnostics and analogs across mechanisms, assay design, and translation.

Qu and colleagues (Contribution 1) examine RNA-modification mechanisms, showing that the repair and translesion-associated DNA polymerases (pol β and pol η) can incorporate m^6^ATP into RNA during RNA gap-filling on RNA–DNA hybrids, yielding unscheduled m^6^A. They also detect m^6^ATP in human cells and report an association with 8-oxoGTP, suggesting that oxidative stress may favour this off-pathway deposition. For assays that use m^6^A as a diagnostic marker, these findings underscore the need for proper controls and orthogonal chemistries to discriminate biologically regulated m^6^A from incorporation-driven artefacts.

Addressing a key challenge to diagnostic reliability, antigenic evolution in SARS-CoV−2, Kim and co-workers (Contribution 2) report universal aptamers that bind spike proteins from multiple variants (D614G-Wuhan-Hu−1, Delta, Omicron). Through consecutive protein-based SELEX and virus-based *viro*-SELEX, they develop a sensitive lateral-flow assay and an ultrasensitive molecular platform that incorporates a rapid PCR step while eliminating reverse transcription, thereby shortening time-to-result without sacrificing sensitivity. These sequence-defined synthetic reagents support detection that preserves performance across circulating variants and accelerates time-to-result for point-of-care workflows.

Noh and colleagues (Contribution 3) address the challenge of input authenticity by developing a COI-barcode-derived SCAR PCR assay to verify *Mantidis Ootheca* at the species level and detect adulteration. Using cytochrome-c-oxidase I barcoding to design species-specific SCAR markers, they implement single- and multiplex-PCR workflows that perform reliably in low-template and mixed-DNA conditions.

By enabling molecular traceability at source, this strategy strengthens supply chain integrity, supports routine quality control, and can be extended to other animal-derived materials used in clinical and research settings.

Design choices in nuclease-based platforms are increasingly informed by computation. Zhu and colleagues (Contribution 4) introduce CrnnCrispr, an interpretable deep-learning framework for predicting CRISPR/Cas9 sgRNA on-target activity. The model yields position-dependent sequence features that can be inspected alongside the predictions, providing design guidance that shortens guide selection cycles and makes optimization decisions auditable. Such transparent predictors are well aligned with diagnostic development, where reproducibility and explainability are central to method validation.

Finally, materials science intersects with DNA damage biology in a comprehensive review by Pascucci and colleagues (Contribution 5) on carbon dots (CDs) for nucleic-acid-centered diagnostics and therapeutics, with particular attention to oxidative DNA damage such as 8-oxo-dG. By linking surface functionalization to photophysics, biocompatibility, and pro-/antioxidant behaviour, the authors provide practical guidance for designing fluorescent readouts and delivery strategies that remain stable in redox-stressed environments, preserving signal fidelity and nucleic-acid integrity.

Taken together, these papers indicate where the field is moving. Assay performance is increasingly shaped by co-design across chemistry, biology, and computation, while challenges such as variant evolution, matrix heterogeneity, and guide efficiency are best addressed by integrating molecular engineering with data-driven design.

In translational contexts, robustness and reproducibility often outweigh record sensitivity: universal aptamers, barcode-based authentication, and interpretable modelling exemplify approaches that maintain performance as samples and targets vary. Finally, because biological systems can follow non-canonical routes, mechanistic vigilance is essential; the observation of unscheduled m^6^A underscores the need for controls and orthogonal chemistries that detect or tolerate such events.

Looking ahead, progress will rely on closing the loops between computational models and experimental benches, enabling interpretable algorithms for guides, primers, and aptamers to directly inform synthesis and assay workflows, ultimately improving limit-of-detection and specificity under realistic sample conditions.

Variant-agnostic recognition layers can be combined with ratiometric or internal-standard strategies to stabilize readouts across matrices, while nanomaterial-enabled tests will benefit from harmonized reporting, surface groups, ζ-potential, photostability, and redox behaviour, to accelerate comparison and regulatory acceptance. For modification-aware assays, control architectures that detect off-pathway marks, such as unscheduled m^6^A, should become standard practice rather than an afterthought.

## References

[1] Marchesini M., Costantino M.L., Raia L., Bono N., Candiani G. (2025). Point-of-Care Nucleic Acid Detection: From Molecular Design to Clinical Reality. ACS Omega.

[2] Moccia M., Antonacci A., Saviano M., Caratelli V., Arduini F., Scognamiglio V. (2020). Emerging technologies in the design of peptide nucleic acids (PNAs) based biosensors. TrAC Trends Anal. Chem..

[3] Veedu R.N., Wengel J. (2010). Locked Nucleic Acids: Promising Nucleic Acid Analogues for Therapeutic Applications. Chem. Biodivers..

[4] O’Sullivan C.K. (2002). Aptasensors—The future of biosensing?. Anal. Bioanal. Chem..

[5] Kudłak B., Wieczerzak M. (2020). Aptamer-based tools for environmental and therapeutic monitoring: A review. Crit. Rev. Environ. Sci. Technol..

[6] Li Y., Lu Y. (2009). Functional Nucleic Acids for Analytical Applications.

[7] Gootenberg J.S., Abudayyeh O.O., Kellner M.J., Joung J., Collins J.J., Zhang F. (2017). Nucleic acid detection with CRISPR–Cas13a/C2c2. Science.

[8] Masi A., Antonacci A., Moccia M., Frisulli V., De Felice M., De Falco M., Scognamiglio V. (2023). CRISPR-Cas assisted diagnostics: A broad application biosensing approach. TrAC Trends Anal. Chem..

[9] Roundtree I.A., Evans M.E., Pan T., He C. (2017). Dynamic RNA Modifications in Gene Expression Regulation. Cell.

[10] Zaccara S., Ries R.J., Jaffrey S.R. (2019). Reading, writing and erasing mRNA methylation. Nat. Rev. Mol. Cell Biol..

[11] Wu X., Zhang Y. (2017). TET-mediated active DNA demethylation: Mechanism, function and beyond. Nat. Rev. Genet..

